# Effects of Different Doses of Fructooligosaccharides (FOS) on the Composition of Mice Fecal Microbiota, Especially the *Bifidobacterium* Composition

**DOI:** 10.3390/nu10081105

**Published:** 2018-08-16

**Authors:** Bingyong Mao, Jiayu Gu, Dongyao Li, Shumao Cui, Jianxin Zhao, Hao Zhang, Wei Chen

**Affiliations:** 1 State Key Laboratory of Food Science and Technology, Jiangnan University, Wuxi 214122, China; maobingyong@jiangnan.edu.cn (B.M.); gujiayu2018@126.com (J.G.); 7130112040@vip.jiangnan.edu.cn (D.L.); cuishumao_053@163.com (S.C.); jxzhao@jiangnan.edu.cn (J.Z.); zhanghao@jiangnan.edu.cn (H.Z.); 2 School of Food Science and Technology, Jiangnan University, Wuxi 214122, China; 3 National Engineering Research Center for Functional Food, Jiangnan University, Wuxi 214122, China; 4 Beijing Innovation Center of Food Nutrition and Human Health, Beijing Technology and Business University (BTBU), Beijing 100048, China

**Keywords:** Fructooligosaccharides, 16S rDNA metagenomic sequencing, intestinal microbiota, *Bifidobacterium pseudolongum*

## Abstract

Fructooligosaccharides (FOS) are a well-known class of prebiotic and are considered to selectively stimulate the growth of bifidobacteria in the gut. Previous studies focused on the growth stimulation of *Bifidobacterium*, but they did not further investigate the bifidobacterial composition and the specific species that were stimulated. In this study, mice were fed with FOS in different doses for four weeks and the composition of fecal microbiota, in particular *Bifidobacterium*, was analyzed by sequencing the V3–V4 region and the *groEL* gene on the MiSeq platform, respectively. In the high-dose group, the relative abundance of Actinobacteria was significantly increased, which was mainly contributed by *Bifidobacterium*. At the genus level, the relative abundances of *Blautia* and *Coprococcus* were also significantly increased. Through the *groEL* sequencing, 14 species of *Bifidobacterium* were identified, among which *B. pseudolongum* was most abundant. After FOS treatment, *B. pseudolongum* became almost the sole bifidobacterial species (>95%). *B. pseudolongum* strains were isolated and demonstrated their ability to metabolize FOS by high performance liquid chromatography (HPLC). Therefore, we inferred that FOS significantly stimulated the growth of *B. pseudolongum* in mice. Further investigations are needed to reveal the mechanism of selectiveness between FOS and *B. pseudolongum*, which would aid our understanding of the basic principles between dietary carbohydrates and host health.

## 1. Introduction

The intestinal tract is an important habitat for bacteria; the colon contains more than 10^12^ bacteria per gram [1], which are mainly anaerobic bacteria [2]. Intestinal bacteria play important roles in the well-being of the large intestine [3]. Among the over 1000 species, many are beneficial to human health, while some are harmful, produce toxins, and cause disease [4]. Resistant starch, dietary fiber, and oligosaccharides in food can successfully reach the large intestine, and are used by the intestinal bacteria as a source of energy [5] and essential nutrients [6]. The resulting metabolites, like short-chain fatty acids, are beneficial for intestinal health.

Bifidobacteria are known to be closely related with intestinal health and are commonly used as health-promoting probiotics [7]. Some carbohydrates in food can escape the digestion and absorption in the stomach and small intestine, and become the main food sources of bacteria in the large intestine. The non-digestible carbohydrates that selectively stimulate the growth of beneficial microbes are called prebiotics, among which the most common are functional oligosaccharides, like lactulose, fructooligosaccharides (FOS), and galactooligosaccharides [8]. In recent years, prebiotics have received much attention and the relationships between prebiotics and the intestinal microbiota have been deeply studied with the aid of high-throughput sequencing techniques. The non-digestible carbohydrates, in particular non-digestible oligosaccharides, have been studied intensely [9]. However, an increasing number of studies have shown that oligosaccharides are not the exclusive substrates for bifidobacteria [10]. It remains unclear how oligosaccharides affect the intestinal microbiota, especially those bacteria with the ability to metabolize oligosaccharides.

FOS are a well-established class of prebiotics and show beneficial health effects to the host. Previous studies using plate counting, quantitative polymerase chain reaction (qPCR), or metagenomic sequencing have demonstrated that FOS selectively stimulated the growth of *Bifidobacterium* [11,12,13]. However, there was no investigation of specifically which bifidobacterial species were stimulated.

Junick et al. developed a method to quantify the human fecal *Bifidobacterium* species using quantitative real-time PCR analysis targeting the *groEL* gene [14], while Hu et al. analyzed the bifidobacterial composition of human and rat gut using the *groEL* gene through high-throughput sequencing on the MiSeq platform [15]. To the best of our knowledge, there are no reports on the bifidobacterial community in mice or its changes upon oligosaccharide treatment.

In this study, mice were fed with FOS in different doses for four weeks and the composition of fecal microbiota was analyzed using MiSeq sequencing to evaluate the effects of FOS treatment. Furthermore, the bifidobacterial communities were analyzed to reveal the relationship between FOS and specific *Bifidobacterium* species.

## 2. Materials and Methods

### 2.1. Chemicals and Reagents

Fructooligosaccharides (GFn, high performance liquid chromatography (HPLC) purity 95.93%) were obtained from Bao Lingbao Biotechnology Co., Ltd. (Shandong, China), the main components of which were 1F-fructofuranosylnystose (2.88%), nistose (32.45%), and 1-kestose (60.60%). AIN-93-VX Vitamin Mix and AIN-93G Mineral Mix were obtained from MP Biomedicals, LLC (Santa Ana, CA, USA).

FastDNA Spin Kit for Soil was obtained from Beijing Lianlixin BioTech Co., Ltd (Beijing, China). QIAquick Gel Extraction Kit was obtained from QIAGEN GmbH (Venlo, The Netherlands). Quanta iTico PicoGreen dsDNA Assay Kit was obtained from USA Life Technologies (Waltham, MA, USA). KAPA Biosystems Library Quantification Kit was obtained from KAPA Biosystems (Woburn, MA, USA). QubitTM dsDNA BR Assay Kit was obtained from Thermo Fisher Scientific (Waltham, MA, USA). TruSeq DNA LT Sample Preparation Kit and MiSeq Reagent Kit were obtained from Illumina Corporation (San Diego, CA, USA).

### 2.2. Animal Experiment Design and Sample Collection

Seven-week-old male C57BL/6J mice were purchased from Shanghai Experimental Animal Center (Shanghai, China). The mice were randomly assigned to three groups, named the control, low-dose, and high-dose groups. Each group contained 10 mice, which were fed in an independent ventilation system (IVC) in the Laboratory Animal Center of Jiangnan University. The conditions of the room were strictly controlled, with a 12 h light/dark cycle and an ambient temperature of 22 °C. The mice were allowed free access to food and water. All of the methods in the animal experiment were reviewed and approved by the ethics committee of Jiangnan University (JN No. 20160927-20161105(67)) and were in accordance with the European Union (EU) guidelines for experimental animals (Directive 2010/63/EU).

**Figure 1:**
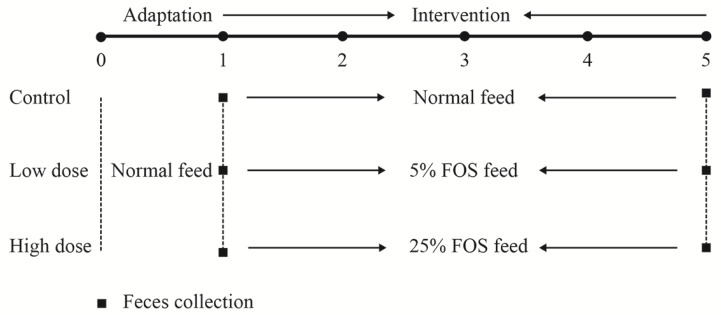
Experimental design. Thirty mice were randomly assigned to three groups, named the control, low-dose, and high-dose groups. During the adaptation period (first week), the mice were fed normal feed. During the intervention period, mice in the control group continued to receive normal feed, while mice in the low- and high-dose groups received the fructooligosaccharides (FOS) feed, containing 5% or 25% FOS (*w*/*w*), respectively. Feces were collected before and after FOS treatment on the first and fifth week.

As shown in Figure 1, the mice were kept for five weeks. The mice in the control group were fed a normal diet according to Campbell’s formula [16] throughout the experiment. The mice in the low-dose and high-dose groups were fed a normal diet during the adaptation period and then modified diets containing 5% or 25% FOS (*w*/*w*), respectively, in the intervention period. The dietary formulae are shown in Table S1. The feed was weighed daily for calculation of FOS intake and the body weight of the mice was recorded every day. The feces were collected according to the methods described by Mao et al. [17] in the first and the last week as shown in Figure 1. The feces were stored at −80 °C for future analysis. At the end of the experiment, the mice were sacrificed and the blood was collected prior to sacrifice. Serum was obtained by centrifugation at 5000 g for 15 min and stored at −80 °C before analysis. The serum biochemical markers were determined by an automatic biochemical analyzer, including glucose, total cholesterol (TC), triglyceride (TG), low-density lipoprotein cholesterol (C-LDL), high-density lipoprotein cholesterol (C-HDL), IgA, IgM, and IgG.

The amount of short-chain fatty acids (SCFAs) in the feces was determined according to the method described by Mao et al. [18]. The SCFAs included acetic acid, propionic acid, butyric acid, isobutyric acid, valeric acid, and isovaleric acid, and the concentration was expressed as μmol/g feces.

### 2.3. Genomic DNA Extraction and PCR Amplification of the V3–V4 Region and the groEL Gene

Bacterial genomes were extracted from the feces using the FastDNA Spin Kit for Soil following the manufacturer’s instructions. To analyze the composition of the fecal microbiota, the V3–V4 region of the 16S rDNA was amplified by PCR with the bacterial genome as the template. The primers were as follows: (forward primer 341F, 5′-CCTAYGGGRBGCASCAG-3′; reverse primer 806R, 5′-GGACTACNNGGGTATCTAAT-3′). The PCR conditions were 95 °C for 5 min; 95 °C for 30 s, 52 °C for 30 s, 72 °C for 30 s, repeat for 30 cycles; and 72 °C for 10 min. To distinguish the species within the genus *Bifidobacterium*, the *groEL* gene was amplified with the primers Bif-groEL-F (5′-TCC GAT TAC GAY CGY GAG AAG CT-3′)/Bif-groEL-R (5′-CSG CYT CGG TSG TCA GGAACA G-3′) [15]. The bacterial genome was set as the template. The samples were distinguished by a barcode consisting of seven bases that were added to the 5′ end of the forward primer 341F or Bif-groEL-F, respectively.

### 2.4. Quantification and Sequencing

The PCR products were recovered from the agarose gel using the QIAquick Gel Extraction Kit and quantified using the QubitTM dsDNA BR Assay Kit according to the manufacturer’s instructions. Libraries were constructed using the TruSeq DNA LT Sample Preparation Kit and sequenced on Illumina MiSeq using the MiSeq v3 Reagent Kit (600 cycles-PE) following the manufacturer’s instructions.

### 2.5. Bioinformatic Analysis

The sequence reads were processed with the QIIME package (Quantitative Insights Into Microbial Ecology). The raw sequences were screened according to the criteria given by Mao et al. [17]. Pair-end reads with an overlap of >10 bp and without mismatches were assembled. Barcodes and sequencing primers were trimmed from the assembled sequences. The operational taxonomic unit (OTU) was established de novo using uclust with 97% sequence identity cutoff. The OTUs of Bif-groEL sequences were taxonomically assigned using the Chaperonin Sequence Database [19] and the OTUs of the V3–V4 region were taxonomically assigned using the Ribosomal Database Project (RDP) Naive Bayes classifier [20]. The first sequence in each OTU cluster was chosen as the representative sequence, and then aligned to the greengenes core set in QIIME using the PyNAST aligner [21,22]. The α- and β-diversity calculations were performed using QIIME and the similarities within the microbial communities were estimated using UniFrac analysis with weighted and unweighted principal coordinate analysis (PCoA) [23].

### 2.6. Isolation and Identification of Bifidobacteria from Mice Feces

To isolate bifidobacteria from the mice feces, fecal samples were collected from the mice in the high-dose group on the fifth week and placed in pre-reduced NaCl solution (0.9% *w*/*v*). The samples were gently dispersed, diluted, plated on Gut Microbiota Medium (GMM) agar [12] containing l-cysteine hydrochloride (0.5 g L^−1^) and mupirocin (0.05 g L^−1^), and incubated in an anaerobic workstation (Whitley DG250, Don Whitley Scientific Limited, West Yorkshire, UK) at 37 °C for 48 h. Colonies were picked up, purified, Gram-stained, and then species-identified by 16S rRNA gene sequencing. The 16S rRNA sequences were deposited in GenBank under the accession number MG820037 to MG820042.

### 2.7. Growth of Bifidobacteria on FOS

The identified bifidobacteria were inoculated on GMM agar containing FOS as the sole carbon source and bromocresol purple as the indicator. Colonies with yellow surroundings on the plates were assumed to be FOS-metabolizing bacteria [12], which was confirmed by high performance liquid chromatography (HPLC) analysis of FOS in the culture supernatant. HPLC was carried out using a Waters 1525 system equipped with a refractive index detector. The separation was performed on an aminopropyl column (250 mm, i.d. 4.6 μm) with a column temperature of 30 °C. The mobile phase was acetonitrile and water (75:25) with a flow rate of 1 mL min^−1^.

### 2.8. Statistical Analysis

The abundance count of different taxa was transformed by log2, and then normalized as follows: From each log transformed measure, the arithmetic mean of all transformed values was subtracted and the difference divided by the standard deviation of all log transformed values for the given sample. After this procedure, the abundance profiles for all samples will exhibit a mean of 0 and a standard deviation of 1 [24]. Analysis of variance (ANOVA) and a *t*-test were used for the comparisons of body weight, serum biochemical markers, SCFAs, Shannon index, and taxa abundance count in different groups (SPSS 16.0). The differences among groups were judged by ANOVA and the differences between two groups were judged by the *t*-test. *p* < 0.05 was considered as significant.

## 3. Results

### 3.1. Body Weight and Dietary Intake

**Figure 2:**
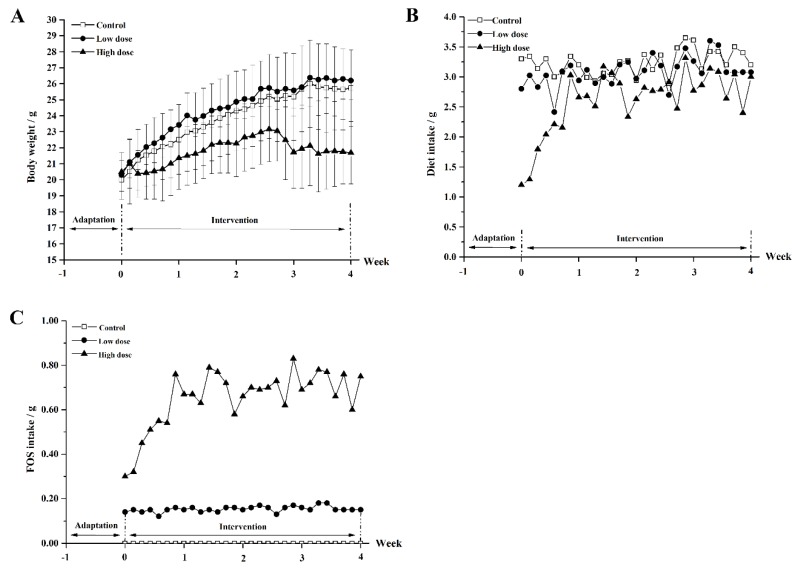
The body weight of mice (**A**), the dietary intake (**B**), and FOS intake (**C**) of mice in the three groups during the intervention period.

The mice in the three groups had a similar initial body weight (Figure 2A). During the intervention period, the body weight of the mice in the control group increased gradually from the initial 20.5 g to the final 25.7 g (*p* < 0.05), and similar growth was observed for the mice in the low-dose group. The average body weight of the mice in the high-dose group was 21.8 g after intervention, which was lower than that in the control and low-dose groups (*p* < 0.01). As shown in Figure 2B, the dietary intakes in the control and low-dose groups were stable at around 3.2 g and 3.1 g, respectively. However, the dietary intake in the high-dose group fluctuated from 1.3 g to 3.3 g with an average of 2.6 g. The FOS intake remained at about 0.15 g in the low-dose group and the FOS intake in the high-dose group varied over time, ranging from 0.33 g to 0.83 g (Figure 2C).

Despite these considerable variations in body weight of the mice after FOS intervention, the serum biochemical markers, including glucose, HDL-C, LDL-C, TC, and TG, showed no significant differences among the three groups (*p* > 0.05, Table 1).

### 3.2. Short-Chain Fatty Acids (SCFAs) Determination in Feces of Mice

The concentrations of six SCFAs in the feces were determined and expressed as μmol/g feces (Table 2). Acetic, propionic, butyric, and isobutyric acid were the dominant SCFAs, and the FOS treatments showed no significant influences on the individual levels of these acids. However, the concentration of total acids in the high-dose group was significantly higher than that in the control and low-dose groups (*p* < 0.05). The percentage of butyric acid in the total acids decreased after FOS treatments.

### 3.3. The Effects of FOS on the Fecal Microbiota of Mice

A total of 1,435,395 high-quality V3–V4 region sequences were generated from 54 samples through MiSeq sequencing, with an average of 26,581 reads per sample. The filtered sequences were clustered with the reference sequences and a 97% identity cutoff was used to define each OTU. The numbers of OTUs ranged from 529 to 3564 for each sample.

**Figure 3:**
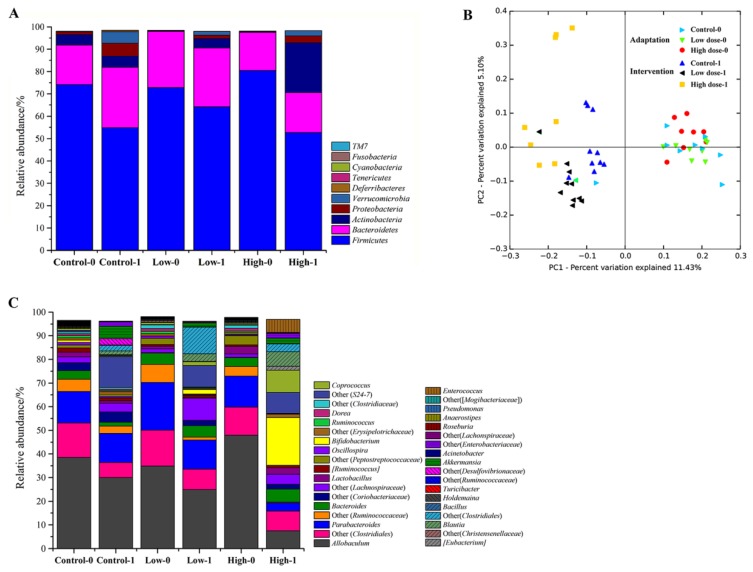
Changes of the composition of fecal microbiota for the two FOS diets. Bacterial diversity at the phylum level (**A**) and genus level (**C**). Principal coordinate analysis (PCoA) score plots based on unweighted UniFrac metrics (**B**), where each point represents the composition of the fecal microbiota of one mouse. “0” stands for before FOS intervention and “1” stands for after FOS intervention.

PCoA showed that the fecal microbiota of the mice in each group were similar during the adaptation period. After intervention, the fecal microbiota changed considerably, even in the control group, indicating that the normal diet also affected the microbiota composition. In Figure 3B, the data of mice microbiota in the three groups are concentrated in the right quadrant and the data are concentrated in the left quadrant after intervention. In addition, the FOS treatment groups can be distinguished from the control group, indicating that FOS intake strongly affected the fecal microbiota.

The bacteria in the mice feces mainly belonged to the phyla Firmicutes, Bacteroidetes, Actinobacteria, and Proteobacteria, among which Firmicutes was the most abundant phylum, accounting for over 70% (Figure 3A). After intervention, the relative abundance of Bacteroidetes, Proteobacteria, and Verrucomicrobia increased and the relative abundance of Firmicutes decreased in the control group. In the low-dose group, there was a significant increase in the relative abundance of Actinobacteria from 0.3% to 4.2% (*p* < 0.05). In the high-dose group, the relative abundance of Actinobacteria was even higher at 22.3%, indicating that the Actinobacteria abundance was positively related to the dose of FOS.

**Figure 4:**
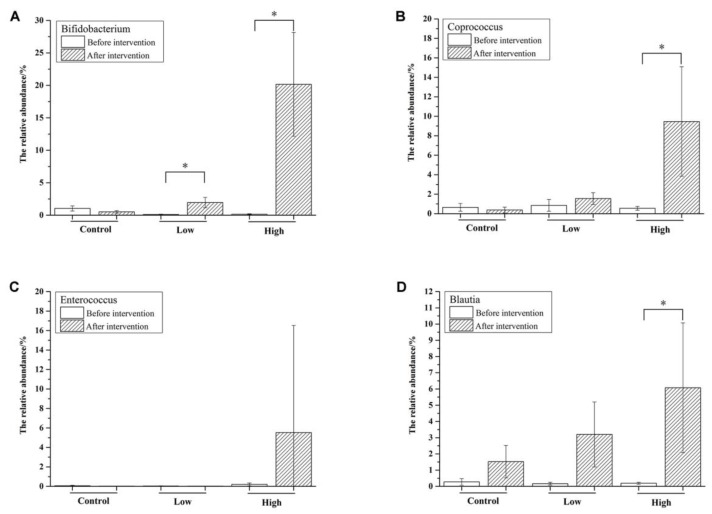
Changes of the relative abundance of selected genera, including *Bifidobacterium* (**A**), *Coprococcus* (**B**), *Enterococcus* (**C**), and *Blautia* (**D**). Significant differences (*p* < 0.05) are indicated with the symbol (*).

Taxonomically, 235 genera belonging to 10 phyla were identified in the mice feces, of which 36 genera with a relative abundance of >0.1% comprised >96% of the microbiota (Figure 3C). During the adaptation period, the most abundant genus was *Allobaculum* (>30%), which belongs to the phylum of Firmicutes. However, its abundance was decreased after intervention, especially in the high-dose group. An unknown genus “Other (S24-7)” belonging to Bacteroidales was significantly increased after FOS intervention compared with that before intervention in all three groups (*p* < 0.05). In the high-dose group, the most obvious change was the significant increase of *Bifidobacterium* from 0.2% to 20.2% (Figure 4A, *p* < 0.01), which contributed most of the increase of Actinobacteria at the phylum level.

Besides *Bifidobacterium*, the relative abundance of *Coprococcus* was also significantly increased (Figure 4B, *p* < 0.05). As shown in Figure 4C, the relative abundance of *Enterococcus* varied a lot among mice in the high-dose group and there was a significant increase in the relative abundance of *Blautia* in the high-dose group (Figure 4D), indicating that the FOS dose had an important effect on the microbiotic composition. As measured by the Shannon index (Table 3), the diversity of the intestinal microbiota was significantly increased in the low-dose group (*p* < 0.05), indicating that FOS intake in low doses acted to broaden bacterial diversity in the mice.

### 3.4. The Effects of FOS on the Composition of Bifidobacterium in Feces

**Figure 5:**
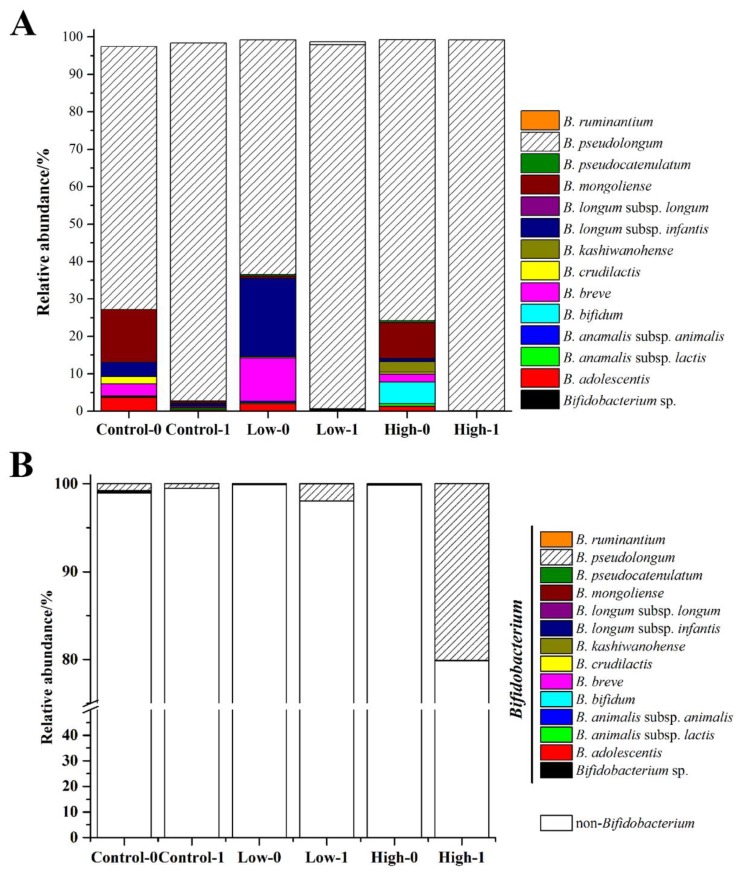
Effects of different doses of FOS on the relative abundance of *Bifidobacterium* species in the *Bifidobacterium* genus (**A**) and in the fecal microbiota (**B**). “0” stands for before FOS intervention and “1” stands for after FOS intervention.

The *groEL* gene was used to study the composition of *Bifidobacterium* at the species level. A total of 1,133,485 sequences were generated from 38 samples, and 14 species/subspecies were detected among all of the samples. As shown in Figure 5A, the *Bifidobacterium* species differed markedly in relative abundance before intervention. *B. pseudolongum*, *B. longum* subsp. *infantis*, *B. breve*, *B. bifidum*, *B. mongoliense*, and *B. adolescentis* were the dominant species, of which *B. pseudolongum* was the most abundant (18–54%). After intervention, *B. pseudolongum* became almost the sole bifidobacterial species, whose abundance increased to 95% in all three groups. The abundance of *B. pseudolongum* reached as far as 98% in the high-dose group. The relative abundances of *Bifidobacterium* in the fecal microbiota were 0.5%, 2.0%, and 20.2% after FOS intervention in the control, low-dose, and high-dose groups (Figure 3C), respectively. Thus, the relative abundance of *B. pseudolongum* reached 19.8% in the fecal microbiota in the high-dose group (Figure 5B), indicating that a high dose of FOS significantly stimulated the growth of *Bifidobacterium* in the gut, especially the *B. pseudolongum* species.

### 3.5. Isolation of B. pseudolongum from Mice Feces and Characterization of Its Growth on FOS

**Figure 6:**
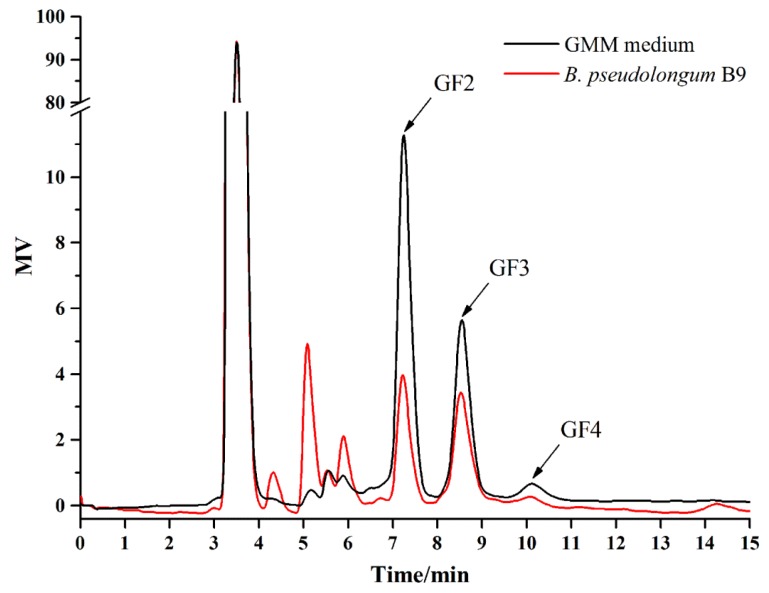
High performance liquid chromatography (HPLC) analysis of the supernatant for *B. pseudolongum* (B9) in Gut Microbiota Medium (GMM)–FOS medium. GF2, GF3, and GF4 were the main components of FOS.

After purification and Gram staining, six strains were identified as *B. pseudolongum* (Table 4), and all of them had the ability to metabolize FOS according to the experiments using GMM agar containing bromocresol purple. The HPLC analysis directly confirmed that *B. pseudolongum* B9 was able to metabolize FOS, especially the components GF2 and GF3 (Figure 6).

## 4. Discussion

In this study, the effects of different doses of FOS on the composition of the intestinal microbiota of mice were investigated using high-throughput MiSeq sequencing. This was not the first and is unlikely to be the last study of the FOS effects on the gut microbiota. During the intervention period, the mice were observed to reduce their dietary intake and their feces became softer, with some feces even becoming sticky in the high-dose group, suggesting that some mice might have excess FOS that leads to slight diarrhea [25,26].

Through the MiSeq sequencing, we were able to analyze the composition of the intestinal microbiota of the mice. Before intervention, Firmicutes and Bacteroidetes were the most abundant phyla, which was in accord with our previous studies [17]. *Allobaculum*, belonging to Firmicutes, was most abundant (>30%) at the genus level in this study. However, in previous studies, *Allobaculum* accounted for <0.1% and *Alitipes*, belonging to Bacteroidetes, was the most abundant genus (>30%) [17].

Diet can alter the gut microbiome within 24 h and these rapid changes are reproducible [27]. Dietary FOS has a modulating effect on the fecal microbiota of the host [28,29,30], which has always been studied using bifidobacteria as the target bacteria. *Bifidobacterium* includes 48 species [31], but not all of them can metabolize FOS. In this study the relative abundance of *Bifidobacterium* was significantly increased after FOS treatment, especially in the high-dose group. We further investigated the composition of *Bifidobacterium* at the species level. There were 14 species/subspecies in the mice feces, dominated by *B. pseudolongum*, *B. longum* subsp. *infantis*, *B. breve*, *B. bifidum*, *B. mongoliense*, and *B. adolescentis*. This appears to be the first description of the composition of *Bifidobacterium* in mice. Hu et al. [15] found that the predominant *Bifidobacterium* species were *B. pseudocatenulatum*, *B. adolescentis*, and *B. longum* in adult human feces, while the dominant species in the gut of rats was *B. animalis* (>90%), suggesting that the composition of *Bifidobacterium* varies substantially among different hosts. It is well known that FOS can selectively stimulate the growth of bifidobacteria in hosts. However, few studies have investigated which species of *Bifidobacterium* were specifically stimulated. In this study, we found that after FOS treatment, the relative abundance of *B. pseudolongum* was significantly increased (>95%), while the other 13 species accounted for only a very small percentage. As shown in Figure 3C, the relative abundance of *Bifidobacterium* at the genus level reached 20% in the high-dose group, nearly all of which was contributed by *B. pseudolongum* (Figure 5A). *Bifidobacterium* species differ in a number of ways, and *B*. *pseudolongum* is particularly versatile in nutrition. As reported by Hopkins et al. [32], *B. pseudolongum* showed higher specific growth rates and bacterial cell yields than *B. longum*, *B. bifidum*, *B. infantis*, *B. catenulatum*, and *B. adolescentis* in a medium with FOS as the sole carbon source. We succeeded in isolating the *B. pseudolongum* strains from the mice feces and verified their abilities to metabolize FOS. Thus, we inferred that FOS significantly stimulated the growth of *B. pseudolongum* in mice.

Besides *Bifidobacterium*, the gut contains many other genera capable of utilizing FOS, such as *Clostridium*, *Streptococcus*, *Coprococcus*, and *Enterococcus* [12,33,34,35]. In this study, *Clostridium* and *Streptococcus* remained at a very low level (<0.1%), while *Coprococcus* was significantly increased in the high-dose group (*p* < 0.05) after FOS treatment (Figure 4B). *Olsenella*, a genus belonging to Actinobacteria, has been shown to have the ability to utilize FOS [17]. Previously, *Olsenella* was significantly increased to a high level, comparable with *Bifidobacterium*, after FOS treatment in high doses. However, *Olsenella* was not detected in the present study. *Blautia*, in the family Lachnospiraceae, is a particularly important microbial genus for nutrient assimilation [36] and has been shown to supply energy to the host via polysaccharide degradation [37,38]. Moreover, through an earlier BLASTP search, *Blautia* was predicted to contain genes related to FOS transport and hydrolysis [12]. Therefore, we propose that *Blautia* may have the ability to utilize FOS, leading to the increase in its abundance herein (Figure 4D).

In this study, we found that FOS significantly increased the abundance of *Bifidobacterium* in mice microbiota. Although a total of 14 *Bifidobacterium* species are known in mice, the increase in this genus was almost entirely contributed by *B. pseudolongum*. We isolated the *B. pseudolongum* strains from mice feces, but the mechanism for the selective stimulation remained unclear. We speculate that the location of these bacteria within the gut, the extent of their access to FOS in the gut, and their inherent abilities to metabolize FOS may be responsible for the selectiveness. It is likely that many species capable of metabolizing FOS could in future be isolated from mice feces. Further in vitro and in vivo investigations are needed to reveal the mechanism of the selective stimulation of *B. pseudolongum* by FOS. This would enable a deeper understanding of the basic principles of dietary carbohydrates and gut microbiota and the development of guidelines to promote health.

## Figures and Tables

**Table 1:** Serum biochemical markers in mice. Glucose (Glu), total cholesterol (TC), triglyceride (TG), low-density lipoprotein cholesterol (LDL-C), high-density lipoprotein cholesterol (HDL-C).

Group	Glu	HDL-C	LDL-C	HDL-C/LDL-C	TC	TG
Control	4.61 ± 0.77	3.06 ± 0.75	0.38 ± 0.09	8.15 ± 0.88	3.45 ± 0.91	1.14 ± 0.28
Low-dose	7.73 ± 2.14	3.28 ± 0.24	0.40 ± 0.04	8.26 ± 0.92	3.72 ± 0.31	1.03 ± 0.47
High-dose	5.77 ± 1.95	2.47 ± 0.25	0.35 ± 0.13	7.81 ± 2.54	2.83 ± 0.38	0.70 ± 0.18
*p* ^1^	NS	NS	NS	NS	NS	NS

^1^ NS represents that the difference is not significant (*p* > 0.05).

**Table 2:** Concentration of short-chain fatty acids (SCFAs) in feces of mice in different groups.

SCFAs, μmol/g ^1^	Control	Low-Dose	High-Dose	*p* ^2^
Acetic acid	36.46 ± 10.83	25.61 ± 15.20	45.24 ± 20.02	NS
Propionic acid	24.37 ± 7.90	17.55 ± 6.92	31.25 ± 9.44	NS
Butyric acid	20.07 ± 7.46	7.43 ± 5.67	13.78 ± 12.24	NS
Isobutyric acid	19.74 ± 6.17	14.45 ± 7.08	31.08 ± 15.81	NS
Isovaleric acid	14.53 ± 5.36	10.45 ± 3.93	18.58 ± 5.17	NS
Valeric acid	10.25 ± 3.60	7.17 ± 2.90	16.52 ± 9.94	NS
Total	125.41 ± 41.23 ^bc^	80.64 ± 28.38 ^bc^	156.44 ± 51.20 ^a^	<0.05

^1^ SCFAs concentration is expressed as μmol/g; ^2^ NS represents that the difference is not significant (*p* > 0.05); abc are different letters that indicate significant differences between the different groups (*p* < 0.05).

**Table 3:** Shannon Index of three groups before and after intervention.

Group	Control			Low-dose			High-dose		
	0	1	*p*	0	1	*p*	0	1	*p*
Shannon Index (SI)	5.29 ± 1.49	5.37 ± 0.35	>0.05	4.99 ± 0.64	5.60 ± 0.51	<0.05	4.32 ± 0.68	5.01 ± 0.75	>0.05

‘0’ stands for before fructooligosaccharides (FOS) intervention; ‘1’ stands for after FOS intervention.

**Table 4:** The identified strains isolated from mice feces.

No.	Strain	Most Positive Match	Ident (%)	GenBank
1	B9	*Bifidobacterium pseudolongum* PV8-2	99%	MG820037
2	B11	*Bifidobacterium pseudolongum* strain UMB-MBP-01	99%	MG820038
3	B24	*Bifidobacterium pseudolongum* strain UMB-MBP-01	99%	MG820039
4	B29	*Bifidobacterium pseudolongum* strain UMB-MBP-01	99%	MG820040
5	B72	*Bifidobacterium pseudolongum* strain UMB-MBP-01	99%	MG820041
6	B129	*Bifidobacterium pseudolongum* PV8-2	99%	MG820042

## Supplementary Materials

The following are available online at http://www.mdpi.com/2072-6643/10/8/1105/s1. Table S1: Dietary formula (g).
