# Supplementary material for: Effects of Different Doses of Fructooligosaccharides (FOS) on the Composition of Mice Fecal Microbiota, Especially the Bifidobacterium Composition
Source: Nutrients. 2018 Aug 16;10(8):1105. doi: 10.3390/nu10081105 (PMC6115998; doi:10.3390/nu10081105)
Supplement: Supplementary file 1 [file nutrients-10-01105-s001.pdf]

Supplementary Materials

Table S1. Dietary formula (g). FOS—fructooligosaccharides.

Formula	Control group	Low-dose group (FOS 5%)	High-dose group (FOS 25%)
	100g Dry matter		
Casein hydrolysate	19.7	19.7	19.7
L-Cystine	0.3	0.3	0.3
Flour	30	30	30
Glucose	34.75	29.75	9.75
FOS	0	5	25
Cocoanut oil	7.88	7.88	7.88
Soybean oil	2.62	2.62	2.62
Choline bitartrate	0.25	0.25	0.25
Vitamin mix	1	1	1
Mineral mix	3.5	3.5	3.5
